# Pregnancy-unrelated spontaneous rupture of a right ovarian artery aneurysm

**DOI:** 10.1016/j.radcr.2021.07.086

**Published:** 2021-08-26

**Authors:** Kumiko Wada, Shigeaki Aoyagi, Yasuo Matsuura, Shogo Urabe, Shin-ichi Nata, Satoru Tobinaga, Hiroshi Yasunaga

**Affiliations:** a Department of Cardiovascular Surgery, St. Mary's Hospital, Kurume, Japan; b Department of Radiology, St. Mary's Hospital, Kurume, Japan; c Department of Emergency and ICU, St. Mary's Hospital, Kurume, Japan

**Keywords:** Ovarian artery aneurysm, Spontaneous rupture, Transcatheter arterial embolization

## Abstract

Spontaneous rupture of an ovarian artery aneurysm is extremely rare. It can lead to retroperitoneal hemorrhage that is often life-threatening. We report a case of pregnancy-unrelated spontaneous rupture of a right ovarian artery aneurysm in a multiparous woman. A 29-year-old woman, gravida 3, para 3, whose latest pregnancy involved uneventful gestation and delivery 2 years previously, was admitted for right flank pain. The urine test result for pregnancy was negative. Computed tomography revealed a large retroperitoneal hematoma and right ovarian artery aneurysm with contrast extravasation. After selective angiography, embolization of the right ovarian artery was successfully achieved using microcoils. Diagnostic angiography with subsequent transcatheter arterial embolization is an effective and less invasive technique for the management of ovarian artery aneurysm.

## Introduction

Although spontaneous rupture of an ovarian artery aneurysm (OAA) is extremely rare, it can lead to retroperitoneal hemorrhage that is often life-threatening [1]. Most cases of spontaneous rupture of an OAA are related to pregnancy and occur during the peripartum or postpartum period. However, spontaneous rupture of an OAA unrelated to pregnancy has been described in a few reports [1].

Recently, transcatheter arterial embolization (TAE) has been increasingly performed to manage ruptured OAA; however, various treatment options, including observation and surgical repair, are available [1,2].

In this paper, we report a case of pregnancy-unrelated spontaneous rupture of an OAA in a young multiparous woman, successfully managed with TAE, and discuss the surgical intervention for this pathology.

## Case report

**Fig. 1:**
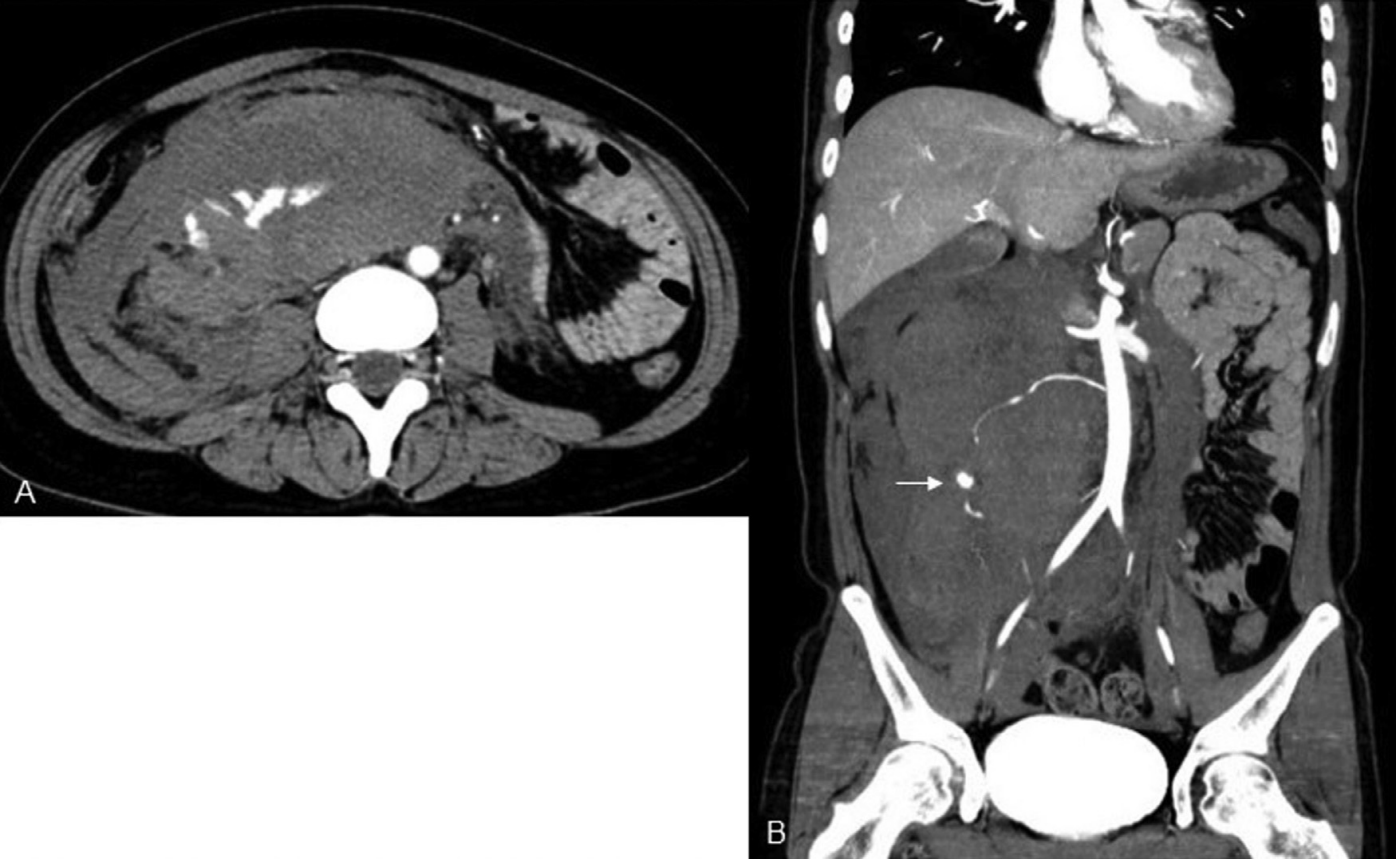
Preoperative computed tomography shows a large retroperitoneal mass with extravasation of contrast extending from the inferior pole of the right kidney to the right pelvic regions (A) and the right ovarian artery with an aneurysm (white arrow) at its middle portion in the center of the mass (B).

**Fig. 2:**
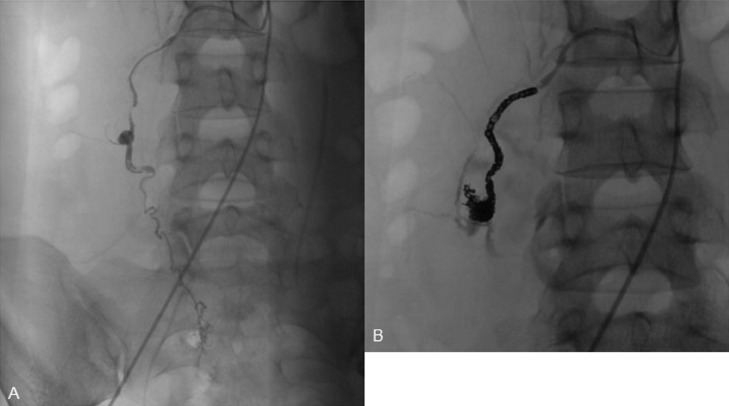
Transfemoral selective angiography shows a tortuous and segmentally dilated right ovarian artery with the saccular aneurysm at its middle portion (A). Postembolization angiography shows complete occlusion of the artery and the disappearance of extravasation of the contrast medium (B).

**Fig. 3:**
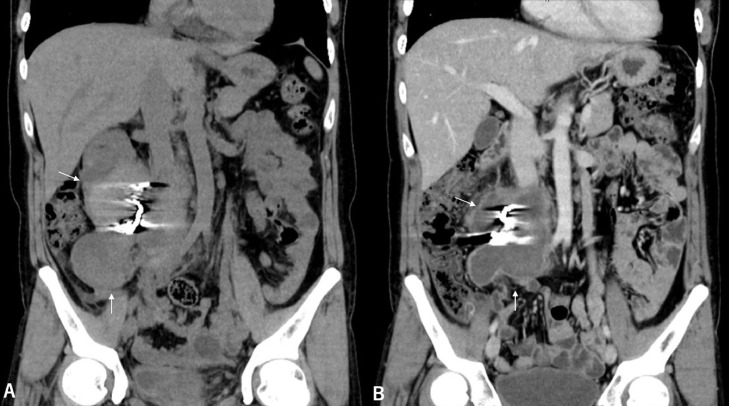
Postoperative computed tomography (11 days (A) and 38 days (B) after TAE) shows a shrinkage of the retroperitoneal hematoma. (White arrow).

A 29-year-old woman, gravida 3, para 3, was transferred to the emergency room for progressively worsening right flank pain that suddenly occurred 2 days previously. The patient had no history of hypertension, gynecologic disease, abdominal surgery, or abdominal trauma. Her last pregnancy was 2 years ago, with an uneventful gestation and delivery. The most recent menstruation period was over 3 weeks prior, with no change in flow and duration. Her family history was negative for genetic or connective tissue disorders. On admission, the patient was alert but anemic. Her blood pressure was 70 of 50 mm Hg, pulse rate 110 beats/min, and body temperature 36.9°C. The abdomen was slightly distended, and palpation revealed tenderness with mild muscle guarding in the right lateral abdomen. Hematological examination revealed a red blood cell count of 305 × 10^4^/mm^3^, a hemoglobin concentration (Hb) of 9.6 g/dL, and a white blood cell count of 15,060/mm^3^. Laboratory data, including coagulation studies, were within the normal ranges. The urine test result for pregnancy was negative. Abdominal ultrasonography revealed a large retroperitoneal mass adjacent to the right kidney. Emergent computed tomography (CT) of the abdomen and pelvis revealed a large retroperitoneal hematoma (10 × 13 cm) with extravasation of contrast extending from the inferior aspect of the right kidney to the right pelvic regions and a right ovarian artery with an aneurysm at its middle portion in the center of the hematoma (Fig. 1). These findings suggest that the retroperitoneal hematoma resulted from a rupture of the right OAA. No other vascular abnormality, such as a left OAA or renal or splenic artery aneurysm, was detected on CT. To identify the origin of the bleeding and manage the bleeding less invasively with TAE, selective angiography of the right ovarian artery was performed immediately after CT under aggressive resuscitation with intravenous infusion of packed red blood cells and crystalloid. Transfemoral selective angiography showed a tortuous and segmentally dilated right ovarian artery with a saccular aneurysm (2.6 × 2.3 cm) (Fig. 2). Subsequently, embolization of the right ovarian artery was performed using microcoils. An angiographic catheter (5Fr, Type Micaelson, Medikit Tokyo) was introduced into the right ovarian artery using an AQUA V-III guiding wire (Cardinal Health Japan, Tokyo). A Carnelian PIXIE ER MSV110S Microcatheter (Tokai Medical Products. Aichi, Japan) was introduced, and then 4 microcoils (C-stopper coil 0.014″ filling coil (Piolax Medical Device, Kanagawa, Japan), Hilal embolization microcoil, Tornado® embolization coil, and Nester® embolization coil (Cook Medical, Bloomington, IN, USA) were placed in the artery proximal and distal to the aneurysm. Postembolization angiography demonstrated complete occlusion of the artery and disappearance of extravasation of contrast (Fig. 2). The patient required a transfusion of 2 units of packed red blood cells to decrease Hb concentration to 8.7 g/dL on postembolization day 1. However, the patient's recovery was uneventful. CT performed on post-embolization days 4, 11, and 38 showed progressive shrinking of the retroperitoneal hematoma without extravasation of contrast (Fig. 3). The patient is well with regular menstruation one year after TAE.

## Discussion

Spontaneous retroperitoneal hemorrhage or hematoma is a life-threatening complication of various clinical pathologies, including vascular diseases, rheumatologic disorders, renal tumors, adrenal tumors, trauma, and coagulopathy. Among vascular diseases, rupture of an abdominal aortic aneurysm and aneurysm of a visceral artery, such as the renal artery or splenic artery, is recognized as the common cause of retroperitoneal hemorrhage. In contrast, spontaneous rupture of an OAA is a rare cause of retroperitoneal hemorrhage [1].

Most cases of spontaneous rupture of an OAA have occurred in late pregnancy or the early postpartum period; however, pregnancy-unrelated rupture of an OAA has also been reported in a few cases [1,3]. According to a literature search using PubMed and Scopus data bases, to the best of our knowledge, 31 patients with spontaneous rupture of an OAA have been previously described in the English literature [1,[3], [4], [5], [6], [7]] (Table 1). Of the 31 cases, 20 (64.5%) were related to pregnancy, and the remaining 11 (35.5%) were not related to pregnancy. However, 29 (96.7%) of 30 patients with a history of pregnancy and delivery were multiparous, with an average parity of 3.7. In addition, aneurysms in the bilateral ovarian arteries have been reported [5], and no OAA has been detected in nulliparous women. Rupture of the OAA in our patient was not associated with pregnancy but occurred in a multiparous woman. These results indicate that aneurysm formation in the ovarian artery and its rupture are closely associated with pregnancy.

**Table 1:** Reported cases of spontaneous rupture of ovarian artery aneurysms.

Case	Age (years)	Gravida/Para	Ova. art	Onset	Treatment
Pregnancy-related rupture of OAA					
1	29	G4P4	L	2d postpartum	laparotomy
2	35	G6P3	L	4d postpartum	laparotomy
3	38	G6P6	R	During delivery	laparotomy
4	32	G3P3	L	4d postpartum	laparotomy
5	35	G3P3	R	4d postpartum	laparotomy
6	26	G5P4	R	1d postpartum	laparotomy
7	23	N/A	R	1m postpartum	laparotomy
8	31	G4P3	R	39w of gestation	laparotomy
9	36	G5P5	R	4d postpartum	TAE
10	38	G3P2	R	during delivery	laparotomy
11	38	G3P2	R	4d postpartum	TAE
12	38	G12P11	R	3d postpartum	laparotomy
13	37	P4	L	39w of gestation	laparotomy
14	30	G5P5	L	5h postpartum	laparotomy
15	39	G5P4	R	5d postpartum	TAE
16	32	P4	L	2d postpartum	laparotomy→TAE
17	37	G4P4	L	4d postpartum	TAE
18	31	G6P4	L	2d postpartum	TAE
19	38	P5	L	4d postpartum	TAE→laparotomy*
20	31	G6P5	R	5d postpartum	TAE
Pregnancy-unrelated rupture of OAA					
21	45	G6P5	L	follicular phase	laparotomy
22	53	G1P1	L	postmenopause	laparotomy
23	55	G2P2	R	postmenopause	TAE
24	46	G3P2	L	2d menstruation	TAE⁎⁎→laparotomy
25	69	G3P3	L	postmenopause	TAE
26	48	G2P2	L	2d menstruation	TAE⁎⁎→laparotomy
27	51	G3P3	R	postmenopause	laparotomy
28	45	G3P3	R	3d menstruation	TAE
29	52	G2P2	R	postmenopause	TAE
30	35	Multigravida	R	?	TAE
31	80	G9P6	L	postmenopause	TAE
Case	29	G3P3	R	luteal phase	TAE

Ova: ovarian, OAA: ovarian artery aneurysm, TAE: transcatheter arterial embolization, d: days, w: weeks, R: right, L: left.

⁎ Laparotomy for hematoma evacuation.

⁎⁎ Failed TAE.

The mechanism of aneurysm formation and/or rupture of an OAA in pregnancy and puerperium is considered to be due to both hemodynamic and hormonal changes. Local fluctuations in blood pressure in the aorta and ovarian artery induced by the enlarging uterus and dilatation of the pelvic arteries in conjunction with the increased uterine blood flow increase the mechanical forces on the vessel walls, leading to local damage. Furthermore, involution of the uterus and the return of other genital organs to the non-pregnant state takes 3-4 weeks postpartum. In addition, high levels of circulating steroid hormones, particularly estrogens, during pregnancy increase pathologic changes in the intima and media of the arterial wall [8]. Consequently, failure of puerperal involution of a segment of the ovarian artery after pregnancy and pregnancy-related changes in the arterial wall may induce subsequent aneurysm formation and/or weakening of pre-existing aneurysms [8]. In our patient, repeated pregnancy may have promoted the development of the aneurysm and caused rupture because she did not have any symptoms or signs of systemic diseases predisposing to aneurysm formation and/or ruptures, such as hypertension or genetic or connective tissue disorders.

CT was useful for arriving at the correct diagnosis of OAA with active bleeding, which caused a retroperitoneal hemorrhage in our patient. Subsequent selective angiography was also helpful in identifying the bleeding location and managing bleeding with TAE. Considering the frequency of rupture at the time of presentation and the uncertain natural history of an OAA, surgical intervention should be performed to prevent further enlargement and rupture of the aneurysm in women of childbearing age and women with a multiparous history when an OAA is identified.

Before 1990, surgical repair (adnexectomy and ligation of the ovarian artery) was mainly performed in all patients; however, TAE has been increasingly employed as the treatment of choice over the past 3 decades [1,3]. TAE was used in 16 (72.7%) of 22 patients with rupture of the OAA after 1990, although 2 patients required surgical repair for failed TAE [1,3]. We also performed TAE of the right ovarian artery using microcoils immediately after identifying the origin of the bleeding using selective angiography. The commencement of treatment with TAE when making the diagnosis of ruptured OAA by selective angiography and less invasiveness of TAE, compared with surgical repair, is a major advantage because most patients with this pathology might be in shock. Furthermore, TAE is applicable when the origin of bleeding is uncertain on CT. However, the optimal treatment strategy for a ruptured OAA is controversial because the patient's condition before treatment varies among patients but is often unstable. Given the promptness and less invasiveness of endovascular treatment, TAE should be considered for patients with a ruptured OAA, particularly those with a stable hemodynamic condition.

Three types of materials, gelatin sponge particles (GSPs), microcoils, and N-butyl-2-cyanoacrylate (NBCA), were used for embolization. A study evaluating TAE outcomes using these materials concluded that although TAE with microcoils required the greatest amount of time, TAE with microcoils or NBCA was more effective and feasible than that with GSPs in terms of primary hemostasis and prevention of recurrent bleeding. Additionally, the study suggested that microcoils were particularly suitable for arresting arterial bleeding arising directly from the main branch of the aorta [9].

Although a case of successful pregnancy following unilateral ovarian artery embolization of a ruptured OAA was reported [10], further studies are required to understand fecundity following unilateral or bilateral ovarian artery embolization.

In conclusion, we report a case of pregnancy-unrelated spontaneous rupture of a right OAA that occurred in a young multiparous patient. Rupture of an OAA should be considered a differential diagnosis in multiparous women complaining of abdominal pain, even in non-pregnant women. Diagnostic angiography combined with subsequent TAE is considered an effective and less invasive technique for managing ruptured OAA.

## Ethics declarations

Ethics approval and consent to participate

The current study was approved by our institutional research ethics board of St. Mary's Hospital (21-0401). Written informed consent was obtained from the patient for the publication of this case report.

## Patient consent

The patient provided informed consent for the publication of her data.
